# Response of VO_2max_ to dark chocolate consumption in healthy subjects: A systematic review and meta‐analysis of randomized controlled trials

**DOI:** 10.14814/phy2.70256

**Published:** 2025-02-25

**Authors:** Aref Mehdipour, Saber Saedmocheshi, Giuseppe Potrick Stefani, Ehsan Amiri, Diako Heidary

**Affiliations:** ^1^ Department of Physical Education and Sport Sciences, Faculty of Humanities and Social Sciences University of Kurdistan Sanandaj Iran; ^2^ Research Group in Olympic Studies (GPEO), School of Health and Life Sciences Pontifical Catholic University of Rio Grande do Sul Porto Alegre Brazil; ^3^ Department of Physical Education and Sport Sciences Allameh Tabataba'i University Tehran Iran

**Keywords:** aerobic exercise, cocoa, exercise training, oxygen consumption

## Abstract

The potential role of dark chocolate (DC) in enhancing exercise performance remains underexplored. While DC has been associated with various health benefits, its specific impact on endurance performance, particularly VO_2max_, has not been conclusively established. This meta‐analysis examined the effect of DC on VO_2max_ in healthy individuals using PRISMA guidelines. Following the application of inclusion and exclusion criteria, five randomized controlled trials (RCTs) involving 144 participants were included, with VO_2max_ as the primary outcome. The meta‐analysis revealed no significant effect of DC on VO_2max_ (SMD = 0.14, 95% CI: −0.16 to 0.44, *p* = 0.36). Heterogeneity among the studies was low (*Q*‐value = 3.34, *I* ^2^ = 0.00, *p* = 0.50), and sensitivity analysis confirmed the robustness of the findings, as excluding individual studies did not alter the results (SMD = 0.14, 95% CI: −0.16 to 0.44, *p* = 0.36). In conclusion, this meta‐analysis suggests that DC consumption does not significantly improve VO_2max_ in healthy individuals. Future research should explore the effects of DC on other aspects of exercise performance, as well as its long‐term impact, to better understand its potential role in athletic and health‐related outcomes.

## 1 INTRODUCTION

Aerobic capacity is recognized as a key criterion in assessing cardiovascular fitness, typically measured through maximum oxygen consumption (VO_2max_). This parameter is important not only for athletes but also for the general population as an indicator of overall health (Wen et al., 2019). Studies have indicated that higher levels of aerobic capacity are associated with improved athletic performance and a reduced risk of cardiovascular diseases, as well as lower mortality rates in non‐athletes (McKendry et al., 2020). Overall, enhancing aerobic capacity can contribute to improved quality of life and better public health (Blair et al., 1996; Kodama et al., 2009; Lee et al., 2010).

Previous studies have demonstrated that engaging in regular physical activity significantly enhances overall fitness levels, which subsequently improves quality of life (Esmail et al., 2020). Furthermore, increasing physical fitness can lead to better health outcomes, including the enhancement of cardiovascular endurance, muscular strength, and functional mobility (Esteban‐Cornejo et al., 2014; Ruiz et al., 2009, 2011). Among these components, considerable attention is given to cardiorespiratory fitness, often referred to as aerobic fitness or maximal aerobic capacity (Ruiz et al., 2011). Cardiorespiratory fitness represents the combined efficiency of the cardiovascular and respiratory systems and reflects the ability to sustain prolonged, intense exercise (Astorino et al., 2012; Brage et al., 2004; Nocon et al., 2008).

To enhance VO_2max_, it is commonly advised to increase exercise training levels (Bouchard et al., 2011). It is approved that sustained aerobic exercise aimed at improving cardiorespiratory endurance can effectively boost VO_2max_ in various populations (Montero et al., 2015). Nevertheless, a recent investigation reveals that for overweight and obese adults, high‐intensity interval training (HIIT) yields greater improvements in VO_2max_ compared to traditional moderate‐intensity continuous endurance training (Bækkerud et al., 2016). Additionally, HIIT has been found to provide significant advantages for individuals with coronary artery disease (Pattyn et al., 2014) and heart failure (Gomes Neto et al., 2018; Haykowsky et al., 2013), as well as for those recovering from a myocardial infarction when prescribed to enhance vascular function.

Cocoa and chocolate are derived from the seeds of Theobroma cacao, a plant traditionally valued for its edible products for thousands of years (Kerimi & Williamson, 2015). In recent decades, dark chocolate (DC) has gained attention as a potent source of polyphenolic antioxidants, which have been shown to exert beneficial effects on various health conditions, including coronary artery disease, hypercholesterolemia, hypertension, skin disorders, and vascular diseases (Actis‐Goretta et al., 2012).

Dark chocolate, rich in bioactive compounds such as polyphenols, flavanols, and epicatechins, has been shown to exert positive effects on the cardiovascular system. Studies indicate that these compounds enhance the production of nitric oxide (NO), a potent vasodilator, which improves blood flow (Ludovici et al., 2017). Enhanced blood flow, particularly during physical activity, can facilitate better oxygen delivery to active tissues and muscles, indirectly contributing to improvements in maximal oxygen consumption (VO_2max_) (Davison et al., 2012). Furthermore, the flavanols in dark chocolate may improve endothelial function by reducing oxidative stress and inflammation, thereby optimizing blood flow (Schroeter et al., 2006). Table 1 summarizes the nutritional composition of dark chocolate based on reliable sources like the USDA FoodData Central and peer‐reviewed studies for a clear overview (Katz et al., 2011; Lippi et al., 2009).

**TABLE 1:** Nutritional Composition of Unsweetened Cocoa Powder (per 100 g).

Nutrient	Amount	Notes
Macronutrients		
Carbohydrates	57.9 g	Includes 37 g dietary fiber and 1.5 g sugars
Proteins	19.6 g	Contains all essential amino acids
Fats	13.7 g	Saturated: 8.1 g (mainly stearic acid), Monounsaturated: 4.6 g, Polyunsaturated: 0.4 g
Calories	228 kcal	Energy content
Micronutrients		
Magnesium	499 mg	Supports muscle function and energy metabolism
Iron	13.9 mg	Essential for oxygen transport in blood
Potassium	1524 mg	Supports heart and muscle function
Phosphorus	734 mg	Vital for bone health and energy production
Zinc	6.8 mg	Supports immune function and wound healing
Copper	3.8 mg	Involved in iron metabolism and antioxidant defense
Manganese	3.8 mg	Supports bone development and metabolism
Vitamin B2 (Riboflavin)	0.24 mg	Supports energy production
Vitamin B3 (Niacin)	2.2 mg	Important for cellular metabolism
Vitamin K	2.5 μg	Supports blood clotting and bone health
Bioactive compounds		
Polyphenols	~10% of dry weight	Includes flavanols like epicatechin and catechin
Theobromine	~2%–3%	Mild stimulant with cardiovascular benefits
Caffeine	~0.2%	Mild stimulant

The combination of dark chocolate consumption and physical activity may yield synergistic effects. Physical activity independently enhances nitric oxide production and endothelial function (Hambrecht et al., 2000). When dark chocolate, as a rich source of flavanols, is consumed, these compounds may amplify the effects of exercise, further improving blood flow and oxygen delivery (Rimbach et al., 2009). This synergistic interaction could enhance cardiovascular and respiratory efficiency, ultimately leading to improvements in VO_2max_ (Taub et al., 2012). Therefore, combining dark chocolate intake with regular physical exercise may serve as an effective strategy for enhancing athletic performance and cardiovascular health.

In 2016, a study conducted an inquiry into the effect of DC on the cardiovascular system. The aim of this study was to investigate the acute effect of a dose of DC (10 g, 70% cocoa) on blood pressure and heart rate changes (Duarte et al., 2016). Only 10 g of cocoa caused a significant increase in parasympathetic modulation and heart rate changes. These results show that a single dose of dark DC improves HRV and reduces SBP and HR. Taken together, these results reinforce the importance of DC consumption for cardiovascular disease prevention and highlight the fact that low doses can be effective in inducing benefits not seen with white chocolate consumption (Duarte et al., 2016).

The hypothesis of this study roots from whether DC supplementation can improve VO_2max_, which is a measure of cardiovascular fitness. Considering the importance of cardiovascular fitness, the purpose of this systematic review and meta‐analysis is to investigate the effect of cocoa/DC consumption on VO_2max_ changes during exercise tests in healthy subjects.

## 2 MATERIALS AND METHODS

The protocol of this systematic review and meta‐analysis used the methods of the Preferred Reporting Items for Systematic Reviews and Meta‐Analyses (PRISMA) statement (Page et al., 2021). The review was registered with PROSPERO (CRD42024586200) at the University of York, UK. However, no study protocol was published before the initiation of the meta‐analysis.

### 2.1 Eligibility criteria

Eligibility criteria were previously selected to minimize the risk of bias. The inclusion and exclusion criteria followed the PICOS strategy (Population/Intervention/Comparator/Outcomes/Study Design) (Table 2). There were no restrictions on language or publication date. Studies that did not meet the eligibility criteria, review publications, letters, duplicates, and the presence of data used in different studies were excluded.

**TABLE 2:** PICOS strategy of the systematic review.

	Inclusion criteria	Exclusion criteria
Population	Health subjects from 18 to 70 years old	Non‐healthy subjects
Intervention	DC consumption with at least 70% of cocoa powder	No DC consumption or presence of another type of supplementation or medication in addition to DC
Comparator	Subjects who received placebo (maltodextrin, white chocolate or milk chocolate) with lower concentration of cocoa powder	Patients with diseases, undergoing medication, or exposed to pharmacological interventions
Outcomes	VO_2max_	Other parameters than VO_2max_
Study design	Randomized controlled trials	Animal studies, in silico studies, controlled trials, reviews, case reports, letters to editors, comments, etc.

### 2.2 Search strategy and data extraction

Two authors (A.M. and D.H.) independently performed the literature search, study selection, and data extraction. Background search in electronic databases, Web of Science, PubMed, Google Scholar, Scopus, and ScienceDirect; the search was limited to English articles and was performed by the following keywords: (exercise training, exercise performance) OR (dark chocolate, Cocoa Powder, cocoa, and chocolate) OR (oxygen uptake, oxygen consumption, VO_2max_). A manual search of the references sections of the selected articles was also performed to identify other relevant studies (Table 3).

**TABLE 3:** Code lines for search in each database.

Database	Search strategy	Results
Pubmed (MEDLINE)	(“chocolate”[MeSH Terms] OR “chocolate”[All Fields] OR (“cocoa Powder”[MeSH Terms] OR (“cocoa Powder”[All Fields] OR (“cocoa”[MeSH Terms] OR (“cocoa”[All Fields]) AND ((“exercise”[MeSH Terms] OR “exercise”[All Fields]) OR (“training”[MeSH Terms] OR “training”[All Fields] OR “sports” [All Fields] OR “aerobic exercise” [All Fields]) AND ((“Oxygen Consumption”[All Fields] OR “aerobic”[All Fields]))	5
WOS	((chocolate OR dark chocolate OR cocoa) AND (exercise OR exercise training OR performance OR aerobic exercise) AND (oxygen uptake OR VO_2max_ OR aerobic capacity))	295
Scopus	((chocolate OR dark chocolate OR cocoa) AND (exercise OR exercise training OR performance OR aerobic exercise) AND (oxygen uptake OR VO_2max_ OR aerobic capacity))	9
ScienceDirect	((chocolate OR dark chocolate OR cocoa) AND (exercise OR exercise training OR performance OR aerobic exercise) AND (oxygen uptake OR VO_2max_ OR aerobic capacity))	297
Google scholar	((chocolate OR dark chocolate OR cocoa) AND (exercise OR exercise training OR performance OR aerobic exercise) AND (oxygen uptake OR VO_2max_ OR aerobic capacity))	298

### 2.3 Selection and data collection process

**FIGURE 1:**
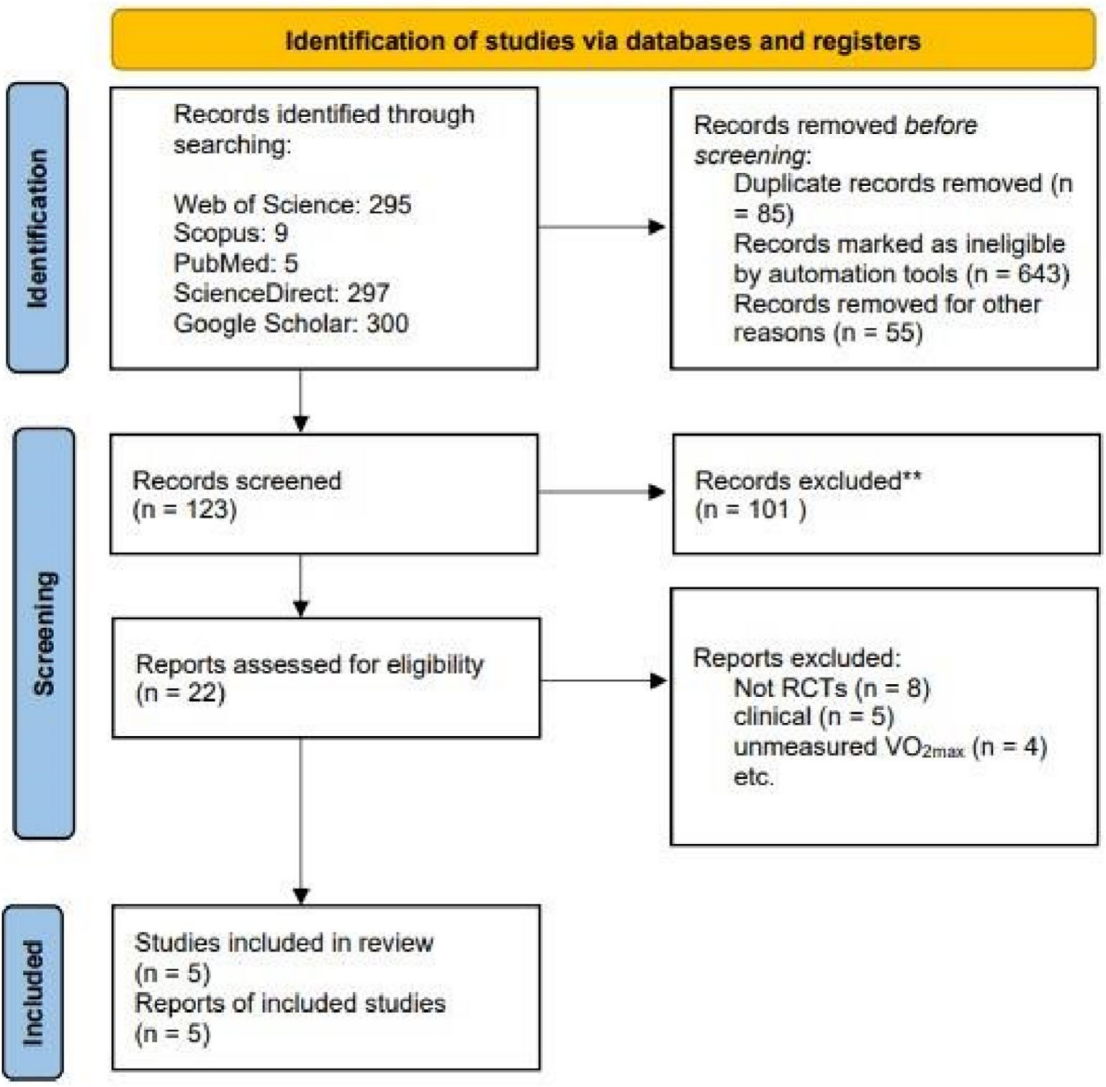
Flowchart of the retrieved articles during the literature search and the studies' selection.

Two authors (A.M. and E.A.) screened all of the citations retrieved independently via a 2‐step process. The initial step consisted of screening titles and abstracts using online software (Rayyan) (Saedmocheshi et al., 2024), and the second step consisted of reviewing full‐text articles to confirm study selection using Foxit Reader software. After each stage, discrepancies were resolved via discussion (Figure 1).

### 2.4 Quality assessment

**FIG 2:**
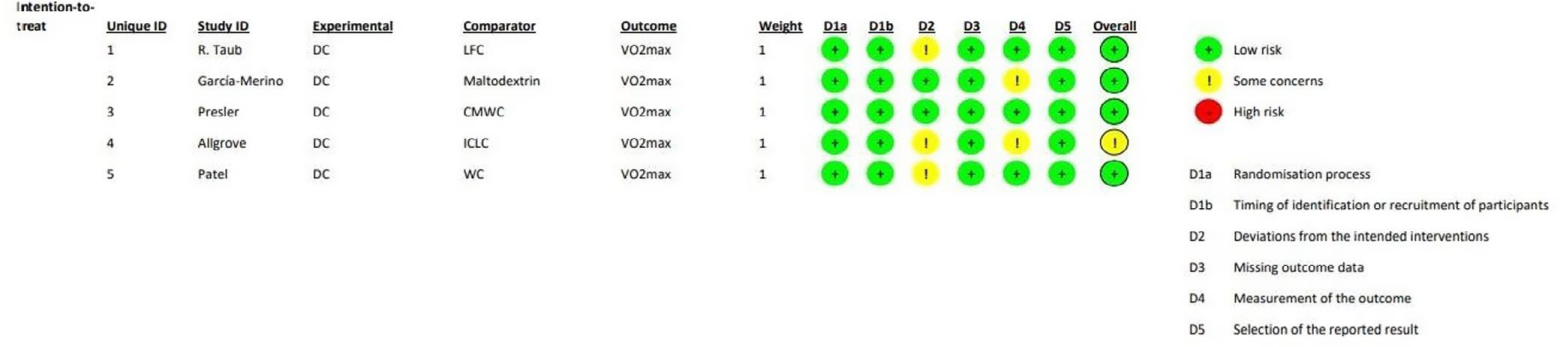
Risk of bias table. CMWC, calorically matched volume of white chocolate; 397 DCFMW, DC flavored molding wafer; ICLC, isocarbohydrate‐fat control cocoa liquor–free chocolate; LFC, Low‐flavanol cocoa; WC, white chocolate.

Cochrane Collaboration's tool (ROB2) was used to independently assess risk of bias (Higgins et al., 2011; Kerimi & Williamson, 2015; Saedmocheshi et al., 2024) in a 2‐step process. This tool considers bias from randomization and blinding (domain 1a), timing of identification or recruitment of participants (domain 1b), deviations in interventions (domain 2), baseline imbalances (domain 2 for parallel group trials), carryover effects (domain 2 for crossover trials), lack of data (domain 3), outcome measurement (domain 4), and bias in reported outcome selection (domain 5). Each domain, as well as the final judgment, was classified as either “low risk of bias,” “some concerns,” or “high risk of bias.” For a study to be classified as “low risk of bias,” it needed to be considered “low risk” in all 5 domains. Studies were classified as “some concerns” when only 1 domain was classified as “some concerns.” If the study had >1 domain assessed as “some concerns,” or ≥1 domain as “high risk,” the overall study classification would be “high risk of bias” (Figure 2) (Silva et al., 2022).

### 2.5 Statistical analysis

Statistical analyses were performed using Comprehensive Meta‐Analysis (CMA) (version 4.0) software. The statistical significance level was set at *p* < 0.05. Furthermore, the standardized mean difference (SMD) effect size index was utilized to examine the impact of DC on VO_2max_. To analyze the studies, it was necessary to determine the disparity between the mean values of VO_2max_ before and after DC consumption, as well as the standard deviation in each study. In all of the studies that were included, the average and standard deviation of VO_2max_ were expressed in terms of mL·kg^−1^ ·min^−1^, except for one study in which VO_2max_ was expressed in terms of L/min that was converted to mL/kg/min (Presler & Webster, 2021). To convert from L/min to mL/kg/min, multiply by 1000 (to convert liter to mL) and divide by the body mass of the subject (kg). The effect size scale utilized to categorize magnitude was as follows: small (<0.2); moderate (0.2–0.5); large (>0.5); and very large effects (>0.8) (Cohen, 2013). The degree of heterogeneity among studies was evaluated through the *Q* test and I2 index, where less than 25%, 25%–75%, and greater than 75% indicated low, moderate, and high heterogeneity, respectively. Publication bias was assessed using funnel plots and Egger's tests, and if funnel plot asymmetry was present, the Trim‐and‐fill analysis was conducted. The Fixed‐effects model was employed in cases of heterogeneity between studies. Additionally, sensitivity analysis was performed to examine whether a particular study or studies influenced the meta‐analysis (Vårvik et al., 2021).

## 3 RESULTS

### 3.1 Literature search strategy

After removing duplicates and screening the titles/abstracts, 22 studies were reviewed for a more detailed evaluation of their full text. After full‐text review, 17 studies were excluded for the following reasons: (1) Unmeasured VO_2max_, (2) were not RCTs, clinical studies, and (Blair et al., 1996) used unhealthy people. A total of 5 studies were included in the current review (Allgrove et al., 2011; García‐Merino et al., 2022; Patel et al., 2015; Presler & Webster, 2021; Taub et al., 2016) (Figure 1).

### 3.2 Study characteristics

The characteristics of the participants are summarized in Table 4. All the studies included in this meta‐analysis were randomized controlled trials. A total of 144 participants were included, and the sample size in each study ranged from 9 to 42. Four of the five included studies included athletes (García‐Merino et al., 2022; Patel et al., 2015; Presler & Webster, 2021); the other studies used sedentary (Saedmocheshi et al., 2024) and healthy individuals (Taub et al., 2016). The participants' ages ranged from 18 to 70 years old. One study was conducted with mixed genders (Taub et al., 2016). In the study of Taub et al. (2016), 17 subjects, 9 of whom were males and 8 females, participated. In addition, the duration of studies varied from 2 to 12 weeks. Additionally, for DC interventions, amounts ranging from 5 to 40 g were considered. The dosage of dark chocolate supplement varied from 70% to 75%.

**TABLE 4:** Summary of included studies.

Author	Study design	Population	Sex	Sample size, treatment/Pla cebo	Dura‐tion (week)	Dark chocolate parameters	Percentage/amount of flavanols and (−)‐epicatechi n in DC	Type, form, commercial supplier supplement/placebo	Result
Taub (2016)	Randomized, double‐blind, placebo‐controlled trial	Sedentary subjects, 40–75 years old	M/F	17, 9/8	12	EXP: 20 g/day of DC PLA: 20 g/day of low‐flavanol cocoa	70%/flavanols: 175.2 mg/20 g epicatechin: 26 mg/20 g	DC, squares of chocolate, The Hershey Company®/DC, The Hershey Company®	NS
García‐Merin (2022)	Randomized, parallel‐group, placebo‐controlled trial	Endurance cross‐cou ntry athletes, 18–50 years old	M	42, 22/20	10	EXP: 5 g/day of CO PLA: 5 g of Maltodextrin	75%/flavanols: 425 mg/20 g	High‐Flavanol Cocoa, Powdered cocoa, Chococru (London, UK)/Maltodextrin, Powder, Prozis (Esposende, Portugal)	NS
Presler (2021)	Randomized, double‐blind, placebo‐controlled trial	Exercise trained, 18–30 years old	F	18, 9/9	4	EXP: 20 g/day of DC PLA: 20 g/day of isocaloric white chocolate	70%/NR	DC, Baking Chunks or Squares, The Hershey Company, Hershey, PA, USA/White Chocolate (WC) Form: Baking Chips Commercial Supplier: The Hershey Company, Hershey, PA, USA	NS
Allgrove (2011)	A single‐blind, randomized, and counterbalanced design	Healthy men, 22 ± 4 years	M	20, 10/10	2	EXP: 40 g of DC twice daily and once 2 hours before exercise PLA: 30.4 g of cocoa liquor–free chocolate	70%/epicatechin: 38.7 mg/40 g M:	DC, Commercial Supplier: Nestlé Noir™ 70% Dark Chocolate/Control Chocolate (CON), Commercial Supplier: Nestlé	NS
Patel (2015)	Randomized controlled crossover study	moderately trained males, 21 ± 1 years	M	9, 9/9	2	EXP: 40 g/day of DC PLA: 40 g/day of white chocolate	75%/NR	DC, Solid form (as chocolate bars), DOVE® Dark Chocolate (Mars, Incorporated, Hackettstown, NJ)/White chocolate, Solid form (as chocolate bars), Milkybar® (Nestlé)	NS

Abbreviations: CO, cocoa; DC, dark chocolate; EXP, experimental; F, female; M, male; NR, not reported; NS, not significant; PAL, placebo.

Previous research has demonstrated that different protocols (e.g., cycling vs. running) may yield varying results in VO_2max_ measurements, particularly in untrained individuals (Poole et al., 2008). In this meta‐analysis, some studies employed cycling protocols, while others used running protocols, which may have influenced the homogeneity of the results. In the study by Presler et al., participants performed a 20 min continuous cycling protocol on a Velotron cycle ergometer. The exercise was divided into two phases: the first 10 min were conducted at a low intensity (50 watts), followed by another 10 min at a moderate intensity (100 watts). This protocol was designed to evaluate physiological responses across different exercise intensities.

Allgrove et al. implemented a cycling‐based exercise protocol to assess the effects of dark chocolate on oxidative stress and metabolic responses. The protocol consisted of 1.5 h of steady‐state cycling at 60% VO₂max (moderate intensity), interspersed with 30‐s high‐intensity bursts at 90% VO₂max every 10 min. Additionally, participants completed a time‐to‐exhaustion trial at 90% VO₂max (high intensity), replicating the demands of competitive cycling.

In the study by García‐Merino et al., male cross‐country athletes underwent a 10‐week polarized endurance training program. The training was structured around three intensity zones based on ventilatory thresholds: approximately 75%–80% of training time was spent in Zone 1 (low intensity, below VT1), 5% in Zone 2 (moderate intensity, between VT1 and VT2), and 15%–20% in Zone 3 (high intensity, at or above VT2). The program also included acute exercise bouts, such as a progressive treadmill test to exhaustion and a 1 km maximal effort run, to evaluate performance adaptations.

Patel et al. employed a cycling protocol to investigate the effects of dark chocolate on physiological and performance outcomes. The protocol included an incremental cycling test to determine VO_2max_ and the gas exchange threshold (GET), followed by 20 min of cycling at 80% GET (moderate intensity) and a 2‐min all‐out sprint (maximal intensity). This design allowed for the assessment of exercise responses across a range of intensities, from moderate to maximal efforts.

Finally, Taub et al. conducted a cardiopulmonary exercise protocol using a stationary bicycle to evaluate aerobic capacity in sedentary individuals (VO_2max_ <30 mL/kg/min). The protocol involved ramped exercise with exhaled gas analysis, performed at a low to moderate intensity, consistent with the participants' sedentary status.

### 3.3 Publication bias

**FIG 3:**
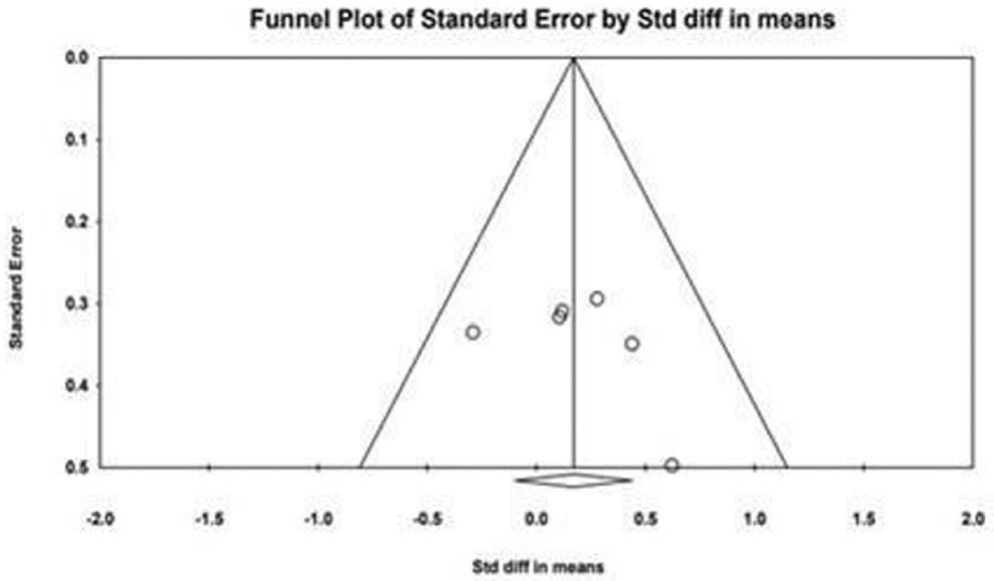
Funnel plot of precision by the difference in means (SMD). Circles represent the SMD for each study, and the diamond represents the pooled SMD.

As shown in Figure 3, the funnel plot was symmetrical and Egger's linear regres162 sion tests provided no evidence for the existence of bias (Egger regression intercept, 3.11 163 [95% CI, −4.89 to 11.13, *p* = 0.30]).

### 3.4 Measurement of VO_2max_

**FIG 4:**
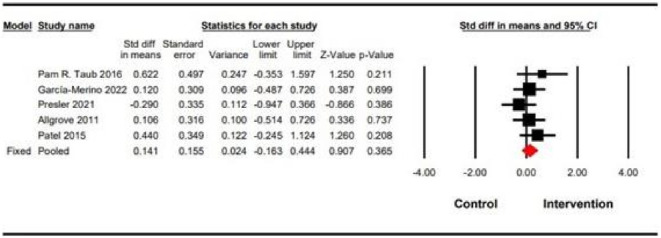
Forest plot for changes in VO_2max_. The black horizontal lines represent the 95% confidence intervals while the squares represent the SMD estimate. The red dia177 mond represents the overall point estimate and 95% confidence intervals from all indi178 vidual studies included in each meta‐analysis. All analyses are based on the fixed‐effects model.

**FIG 5:**
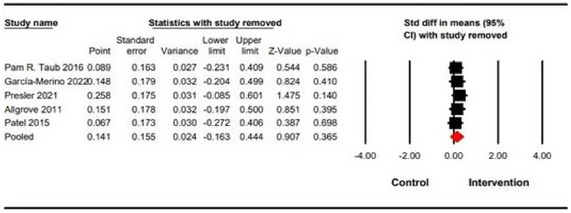
Sensitivity analysis.

Five studies including a total of 144 participants reported VO_2max_ as an outcome measure. In regard to Figure 4, four of the five studies show the change of VO_2max_ towards the intervention group. However, these changes are not statistically significant. According to the findings of this meta‐analysis, DC had no significant effect on VO_2max_ (SMD = 0.14 (95% CI: −0.16 to 0.44), *p* = 0.36) (Figure 4). Analysis of heterogeneity demonstrated insignificant heterogeneity (*Q*‐value = 3.34, *I* ^2^ = 0.00, *p* = 0.50). In addition, the sensitivity of the results was also determined by excluding studies that had a high risk of bias. The sensitivity of the studies was tested by excluding them, but no significant 173 changes were observed in VO_2max_ (SMD = 0.14 (95% CI: −0.16 to 0.44) *p* = 0.36) (Figure 5).

### 3.5 Additional analysis

Regarding subgroup analysis, in athlete individuals, DC did not favor an increment of 186 in VO_2max_ (SMD = 0.225; 95% CI, − 0.269 to 0.778; *p* = 0.340; *I* ^2^ = 13.00%) the overall same 187 effect was not observed in non‐athlete individuals (SMD = −0.082; 95% CI, − 0.291 to 188 0.456; *p* = 0.665; *I* ^2^ = 00.00%) (Table 5).

**TABLE 5:** Subgroup analyses of the effect of DC on VO_2max_.

	*N* (both group)	SMD	95% CI	*p* Value	*p* (difference)
Athlete					
García‐Merino (2022)	42	0.120	(−0.487, 0.726)	0.69	0.66
Presler (2021)	36	−0.290	(−0.947, 0.366)	0.38	
Patel (2015)	9	0.440	(−0.245, 1.124)	0.20	
Non‐athlete					
Taub et al. (2016)	17	0.622	(−0.353, 1.597)	0.21	0.34
Allgrove et al. (2011)	40	0.106	(−0.5014, 0.726)	0.73	

## 4 DISCUSSION

To our knowledge, this is the first systematic review and meta‐analysis of randomized controlled trials characterizing DC consumption on changes in VO_2max_ during exercise tests in healthy subjects. The results of this study demonstrate that chronic consumption of DC can improve VO_2max_ in healthy subjects, but these changes are not statistically meaningful.

Regular exercise training results in various physiological changes that enhance athletic performance (Heinonen et al., 2014; Shaw et al., 2020), and these effects can be further amplified through the use of dietary supplements (Valenzuela et al., 2019). The impact of DC supplementation on exercise performance remains a topic of debate, with mixed findings reported in the literature. For example, some studies indicate that DC and its key flavanols may boost cardiovascular efficiency, endurance, or recovery, while other research suggests no significant benefits or even adverse effects (Kopustinskiene et al., 2015; Moreno‐Ulloa et al., 2018; Schwarz et al., 2018).

Given the existing uncertainty, we have undertaken a systematic review and meta‐analysis of studies investigating the impact of DC or cocoa intervention on the aerobic capacity of healthy individuals. For instance, García‐Merino et al. (García‐Merino et al., 2022) conducted a study involving endurance athletes who were randomly assigned to either a supplement or control group at the onset of their training season. The supplement group consumed cocoa rich in flavanols, while the control group received maltodextrin as a placebo. This intervention was carried out over a period of 10 weeks, during which both groups underwent exercise training. The results indicated that VO_2max_ was improved in both groups of athletes (Page et al., 2021). However, while the control group also demonstrated improvements in maximal aerobic capacity, the cocoa‐supplemented group did not (Vårvik et al., 2021). In addition, in another study, twenty‐eight young male soccer players (18–20 years old) consumed 105 g of flavanol‐containing milk chocolate (FCMC) (168 mg flavanols) or cocoa butter chocolate (CBC) (5 mg flavanols) daily, consumed as part of their normal diet for 14 days. Their evaluation indicated interesting results. These results indicate that the CBC group, which contained less flavanols (5 mg flavanols per 105 g) gave rise to a significant improvement in VO_2max_ compared to the FCMC group, which had more flavanols in their diet (168 mg flavanols per 105 g) (Fraga et al., 2005).

The particular mechanisms by which DC influence physiological function are not clearly defined. A positive health benefit attributed to DC consumption is the potent polyphenol quality of the flavonoid (−)‐epicatechin (Hollman et al., 2011; Yang et al., 2024). Nevertheless, the available evidence indicates that flavonoids possessing a high total antioxidant capacity (TAC) may impede rather than facilitate skeletal muscle adaptation to exercise (Jacobs et al., 2013; McDonald et al., 2021). Taub et al. (2016) investigated the effects of a 20 g/d dose of DC supplementation over a period of 3 months and observed that this dosage produced the most significant outcomes in cultured cells in terms of (−)‐epicatechin blood levels. It is conceivable that a higher dosage of DC may have an adverse impact on physiological function, whereas a lower dosage may not.

Recent studies suggest a potential mechanism by which (−)‐epicatechin, a compound found in dark chocolate (DC), can significantly alter mitochondrial structure and promote mitochondrial biogenesis (Jacobs et al., 2013; McDonald et al., 2021). It has been proposed that (−)‐epicatechin may bind to cell surface G protein‐coupled estrogen receptors, thereby enhancing metabolic regulation and stimulating mitochondrial biogenesis (Valenzuela et al., 2019). Taub et al. (2016) also reported that a 3‐month supplementation of 20 g/day of cocoa rich in (−)‐epicatechin resulted in improved VO_2max_ and cycling power output in a group of sedentary individuals, as well as in middle‐aged men and women. This positive effect was likely attributed to increased mitochondrial efficiency in response to (−)‐epicatechin, as evidenced by a 140% increase in citrate synthase activity, a marker of mitochondrial function (). Previous research on isolated rat muscle also demonstrated that (−)‐epicatechin, either alone or in combination with exercise, could induce structural and metabolic adaptations in both skeletal and cardiac muscle (Vårvik et al., 2021). Furthermore, the findings of Presler et al. suggest that a short‐term (30‐day) moderate intake (20 g/day) of 70% DC could lead to a significant 9.6% increase in resting energy expenditure (REE), indicating potential alterations in mitochondrial function. However, no significant differences in VO_2max_ were observed when compared to the control group (Presler & Webster, 2021).

## 5 CONCLUSIONS

This is the first systematic review and meta‐analysis to investigate the effects of DC on VO_2max_. The findings of this meta‐analysis indicate that the consumption of DC supplements at varying dosages (ranging from 20 to 40 g) and durations (2–12 weeks) do not have a significant impact on VO_2max_. Although the effect of chocolate supplementation on VO_2max_ was not statistically significant (Figure 4), all but one study found changes favoring the supplement group.

This study has several limitations that should be considered when interpreting the results. Firstly, the methods used to measure VO_2max_ across the included studies were heterogeneous, involving both cycling and running protocols, which may have affected the homogeneity of the findings. To enhance comparability, future studies are encouraged to adopt standardized and consistent protocols. Secondly, there was insufficient reporting on the percentage and nutritional composition of the dark chocolate used, and the sample sizes were relatively small, potentially limiting the generalizability of the results. It is recommended that future research explicitly detail the composition of dark chocolate, increase sample sizes, and maintain consistent dosages. Additionally, the influence of factors such as the lifestyle and socio‐economic background of participants was not addressed, which could act as confounding variables. Furthermore, greater attention should be paid to the composition of dark chocolate, including its flavanol, (−)‐epicatechin, and caffeine content, as these components may play a decisive role in the outcomes. Addressing these limitations in future research could significantly improve the quality and reliability of the findings.

## AUTHOR CONTRIBUTIONS

A.M. Conceptualization; methodology; validation; data curation; Data analysis; writing—original draft; writing—review and editing; visualization; resources. S.S. project administration; Data curation; writing—original draft. G.P.S. visualization; Data curation; writing—original draft. Ehsan Amiri: Validation; data curation. Diako Heidary: Validation; writing—original draft.

## FUNDING INFORMATION

No sources of funding were used to assist in the preparation of this article.

## CONFLICT OF INTEREST STATEMENT

Authors declare that they have no conflicts of interest relevant to the content of this review.

## Data Availability

Not applicable.
